# Supplementary material for: Invasive earthworms unlock arctic plant nitrogen limitation
Source: Nat Commun. 2020 Apr 14;11:1766. doi: 10.1038/s41467-020-15568-3 (PMC7156384; doi:10.1038/s41467-020-15568-3)
Supplement: Supplementary file 1 — Supplementary Information [file 41467_2020_15568_MOESM1_ESM.pdf]

SUPPORTING INFORMATION: Invasive earthworms unlock the Arctic plant nitrogen limitation

Authors:

Gesche Blume-Werry[1,2], Eveline J. Krab[3,2], Johan Olofsson[2], Maja K. Sundqvist[2,4], Maria Väisänen[5, 6], Jonatan Klaminder[2]*

[1]Experimental Plant Ecology, Institute of Botany and Landscape Ecology, University of Greifswald, Soldmannstr. 15, 17487 Greifswald, Germany

[2] Climate Impacts Research Centre, Department of Ecology and Environmental Science, Umeå University, Abisko, Sweden

[3]Swedish University of Agricultural Sciences, Department of Soil and Environment, SE-750 07 Uppsala, Sweden

[4]Swedish University of Agricultural Sciences, Department of Forest Ecology and Management, SE-901 83 Umeå, Sweden

[5] Arctic Centre, University of Lapland, P. O. Box 122, FI-96101 Rovaniemi, Finland

[6] Ecology and Genetics Research Unit, University of Oulu, P. O. Box 3000, FI-90014 Oulu, Finland

Supplementary Figures

Supplementary Figure 1. Experimental setup of the mesocosm experiment. Platforms (black rectangles) where mesocsoms with heath (grey rectangles) and meadow (green rectangles) vegetation were installed. Mesocosm ID-numbers are shown within each mesocosm, while numbers in the bottom right corner of each platform represent blocks used for our applied block-designed statistics. Platforms that received worm addition are surrounded by red lines and mesocosms that received labelled litter addition are surrounded by blue lines; other mesocosms received unlabelled litter. Location of loggers recording air temperatures and soil moisture (moist.) are also shown.

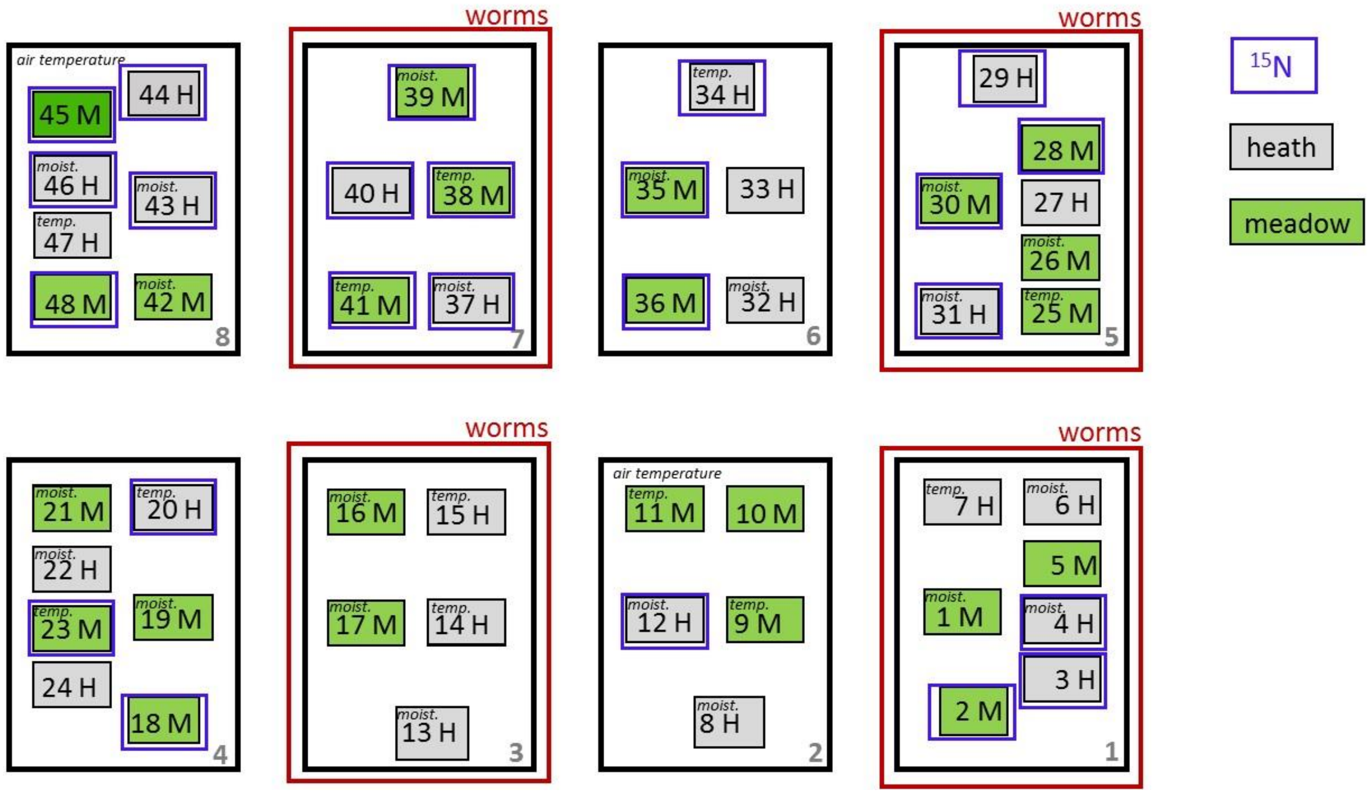

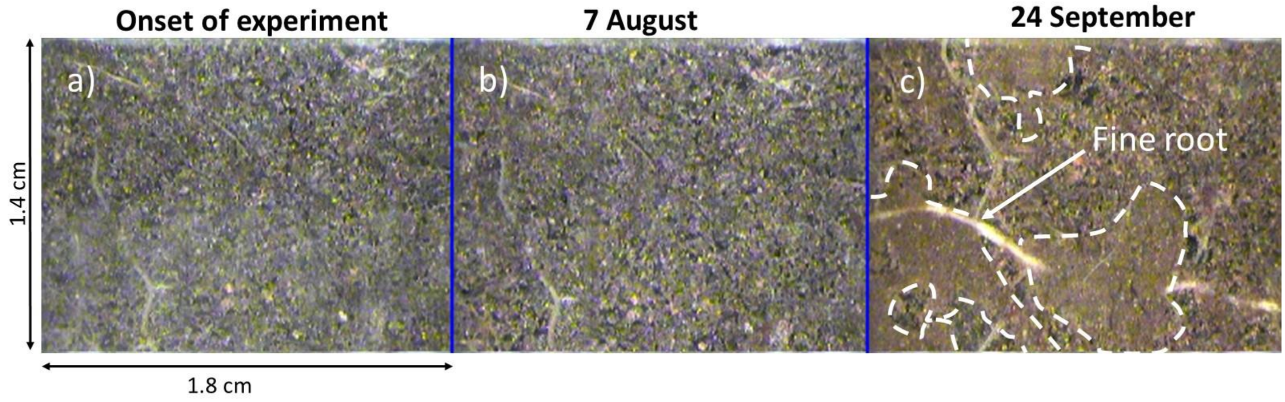

Supplementary Figure 2. **Minirhizotron images illustrating fine root growth and deposition of earthworm casting**. Images from the same area taken in a) June, b) August and c) September. Scale of the images are shown using black arrows. Note how earthworm castings (marked with white dashed lines) have formed between August and September and how considerable fine root (white arrow) growth has occurred during the same time period.

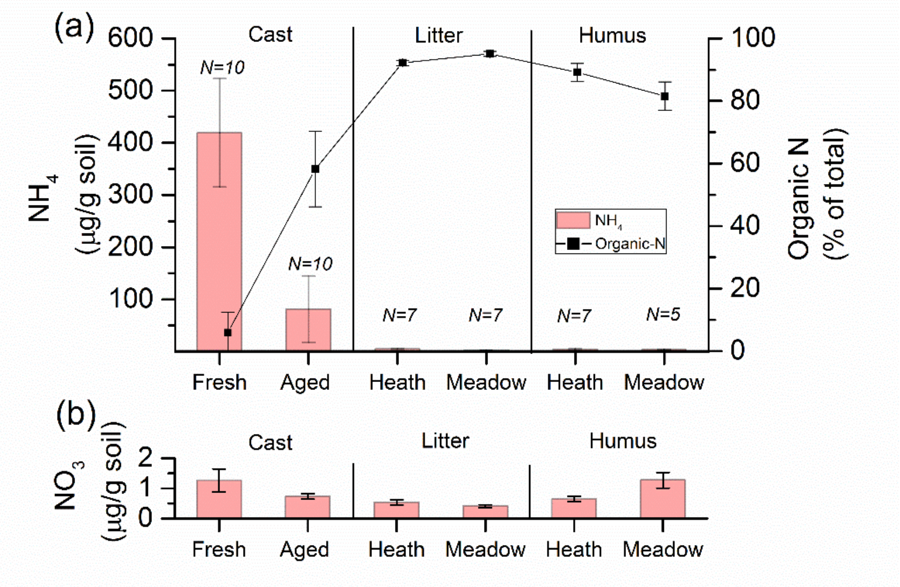

Supplementary Figure 3. **Nitrogen forms in cast, litter and humus.** Data shown include water leachable nitrogen forms from the earthworm invasion gradient at Jiebren described in detail by Wackett et al 2018 [1]. Analyzed matrixes include fresh earthworm casts (<7days old), aged earthworm cast (seasonal age), litter and humus. (a) The concentration of ammonium (NH_4) and proportion of total dissolved nitrogen (± std. err) that are in organic forms in earthworm cast in relation to litter and humus not subjected to earthworms. (b) Dissolved nitrate (NO_3) in the same leachate as above (± std. err).

Supplementary Tables

Supplementary Table 1. **Timeline showing the different activities of the experiment**. Dates of activities (mesocosm manipulations, sampling and measurements) conducted following the installation of the mesocosm in 2013 are shown in the table below.

Time	Activity
Fall-2013	*Heath and meadow monoliths installed, the common garden experiment started*
2013-2017	*Stabilization*
2017-06-09	*1st earthworm inoculation*
2017-06-13	*1st fine root imaging*
2017-06-16	*PRS probes installed*
2017-06-19	*Addition of litter (unlabelled, labelled)*
2017-07-27	*Vegetation & litter survey, NDVI*
2017-08-07	*2nd fine root imaging*
2017-08-18	*Soil sampling for PLFAs*
2017-08-21	*Plant sampling for N &* $\delta^{15}N$
2017-09-25	*3rd fine root imaging*
2017-10-03	*PRS probes collected*
2017-2018	*Winter*
2018-06-16	*2nd earthworm inoculation*
2018-10-08	*Measurement of graminoid height & number of floral shoots*

Supplementary Table 2. **Exchangable nutrients sampled with the Plant Root Simulator probes**. Note that the estimated method detection limit (MDL) for nitrate (NO_3) and ammonium (NH_4) is 2 µg/10 cm^2 and thus, most measurements of NO_3 are below the MDL. Measurements of all other elements are well above the MDL. There were no statistically signficant difference between the treatments for any of the studied nutrient.

	NO3 µg/10cm²		NH4 µg/10 cm²		Ca µg/10 cm²		Mg µg/10 cm²		K µg/10cm²		P µg/10 cm²		Fe µg/10 cm²		Mn µg/10 cm²	
Heath	1.9	±0.5	5.1	±3.5	2073	±187	213	±34	115	±62	11.7	±4.9	3.2	±0.2	53	±29
Heath +W	1.6	±0.3	3.1	±0.7	2152	±214	174	±22	67	±35	5.9	±1.3	4.8	±0.8	21	±12
Meadow	1.5	±0.2	4.3	±0.6	1420	±233	321	±35	306	±97	13.1	±2.1	3.7	±0.9	88	±23
Meadow +W	1.1	±0.1	4.9	±1.0	1331	±195	336	±35	311	±86	14.7	±2.1	3.7	±0.5	89	±25

Supplementary Table 3. **Data used for calculating plant community-N as a function of mesocosms.** The data is arranged in the order of vegetation type, ^{15}N composition of added litter (UL =unlabelled; L=labeled), mesocosm identity (ID) and earthworm additions (N= no additions; Y= additions). The proportional aerial cover (p) of plant functional groups (graminoids, forbs, evergreen and deciduous shrubs) are shown along with their N content (N%). Here, average value of N for each earthworm treatment was used to calculate plant community N and thus, the between mesocosm variability in plant abundance is driving the variation between treatments. Description of how the data was calculation and compiled are found below the table.

VEG. TYPE	^{15}N	ID	WORMS	GRAMIN. P	FORBS P	EVERGR. P	DECID. P	GRAMI. N (%)	FORBS N (%)	EVERGR. N (%)	DECID. N (%)	COMMUNITY N (%)
**HEATH**	UL	8	N	0.48	0.00	0.43	0.10	0.99	2.22	1.15	1.38	1.10
**HEATH**	L	12	N	0.14	0.05	0.77	0.05	0.99	2.22	1.15	1.38	1.18
**HEATH**	L	20	N	0.71	0.19	0.02	0.07	0.99	2.22	1.15	1.38	1.26
**HEATH**	UL	22	N	0.61	0.04	0.24	0.11	0.99	2.22	1.15	1.38	1.12
**HEATH**	UL	24	N	0.08	0.00	0.67	0.25	0.99	2.22	1.15	1.38	1.19
**HEATH**	UL	32	N	0.61	0.08	0.16	0.16	0.99	2.22	1.15	1.38	1.18
**HEATH**	UL	33	N	0.53	0.00	0.47	0.00	0.99	2.22	1.15	1.38	1.06
**HEATH**	L	34	N	0.16	0.32	0.52	0.00	0.99	2.22	1.15	1.38	1.47
**HEATH**	L	43	N	0.00	0.00	0.90	0.10	0.99	2.22	1.15	1.38	1.17
**HEATH**	L	44	N	0.08	0.00	0.40	0.52	0.99	2.22	1.15	1.38	1.25
**HEATH**	L	46	N	0.37	0.04	0.52	0.07	0.99	2.22	1.15	1.38	1.15
**HEATH**	UL	47	N	0.49	0.00	0.51	0.00	0.99	2.22	1.15	1.38	1.07
**MEADOW**	UL	9	N	0.48	0.52	0.00	0.00	1.10	2.22	1.26	1.38	1.69
**MEADOW**	UL	10	N	0.44	0.56	0.00	0.00	1.10	2.22	1.26	1.38	1.73
**MEADOW**	UL	11	N	0.64	0.28	0.08	0.00	1.10	2.22	1.26	1.38	1.43
**MEADOW**	L	18	N	0.38	0.62	0.00	0.00	1.10	2.22	1.26	1.38	1.79
**MEADOW**	UL	19	N	0.31	0.69	0.00	0.00	1.10	2.22	1.26	1.38	1.87
**MEADOW**	UL	21	N	0.60	0.40	0.00	0.00	1.10	2.22	1.26	1.38	1.55
**MEADOW**	L	23	N	0.45	0.55	0.00	0.00	1.10	2.22	1.26	1.38	1.72
**MEADOW**	L	35	N	0.40	0.60	0.00	0.00	1.10	2.22	1.26	1.38	1.77
**MEADOW**	L	36	N	0.37	0.63	0.00	0.00	1.10	2.22	1.26	1.38	1.81
**MEADOW**	UL	42	N	0.68	0.32	0.00	0.00	1.10	2.22	1.26	1.38	1.46
**MEADOW**	L	45	N	0.38	0.63	0.00	0.00	1.10	2.22	1.26	1.38	1.80
**MEADOW**	L	48	N	0.29	0.44	0.26	0.00	1.10	2.22	1.26	1.38	1.64
**HEATH**	L	3	Y	0.59	0.00	0.24	0.16	1.72	2.34	1.29	1.56	1.59
**HEATH**	L	4	Y	0.41	0.00	0.41	0.18	1.72	2.34	1.29	1.56	1.52
**HEATH**	UL	6	Y	0.28	0.00	0.41	0.31	1.72	2.34	1.29	1.56	1.49
**HEATH**	UL	7	Y	0.54	0.00	0.38	0.08	1.72	2.34	1.29	1.56	1.55
**HEATH**	UL	13	Y	0.11	0.15	0.59	0.15	1.72	2.34	1.29	1.56	1.54
**HEATH**	UL	14	Y	0.08	0.16	0.24	0.51	1.72	2.34	1.29	1.56	1.63
**HEATH**	UL	15	Y	0.16	0.03	0.81	0.00	1.72	2.34	1.29	1.56	1.39
**HEATH**	UL	27	Y	0.31	0.00	0.64	0.05	1.72	2.34	1.29	1.56	1.44
**HEATH**	L	29	Y	0.35	0.00	0.53	0.12	1.72	2.34	1.29	1.56	1.47

**HEATH**	L	31	Y	0.49	0.00	0.47	0.05	1.72	2.34	1.29	1.56	1.51
**HEATH**	L	37	Y	0.45	0.04	0.19	0.32	1.72	2.34	1.29	1.56	1.61
**HEATH**	L	40	Y	0.23	0.08	0.35	0.35	1.72	2.34	1.29	1.56	1.56
**MEADOW**	UL	1	Y	0.52	0.48	0.00	0.00	1.52	2.34	1.43	1.56	1.91
**MEADOW**	L	2	Y	0.24	0.76	0.00	0.00	1.52	2.34	1.43	1.56	2.14
**MEADOW**	UL	5	Y	0.43	0.57	0.00	0.00	1.52	2.34	1.43	1.56	1.99
**MEADOW**	UL	16	Y	0.46	0.44	0.10	0.00	1.52	2.34	1.43	1.56	1.87
**MEADOW**	UL	17	Y	0.34	0.66	0.00	0.00	1.52	2.34	1.43	1.56	2.06
**MEADOW**	UL	25	Y	0.58	0.42	0.00	0.00	1.52	2.34	1.43	1.56	1.86
**MEADOW**	UL	26	Y	0.70	0.30	0.00	0.00	1.52	2.34	1.43	1.56	1.77
**MEADOW**	L	28	Y	0.73	0.27	0.00	0.00	1.52	2.34	1.43	1.56	1.74
**MEADOW**	L	30	Y	0.32	0.32	0.36	0.00	1.52	2.34	1.43	1.56	1.75
**MEADOW**	L	38	Y	0.47	0.21	0.00	0.32	1.52	2.34	1.43	1.56	1.71
**MEADOW**	L	39	Y	0.64	0.36	0.00	0.00	1.52	2.34	1.43	1.56	1.82
**MEADOW**	L	41	Y	0.71	0.29	0.00	0.00	1.52	2.34	1.43	1.56	1.76

Supplementary Methods 1.

Species abundance (% cover) as determined by point intercept method was used as weight when calculating weighted plant community N content. The specific data used for the calculation are shown. Community N from other local studies was compiled from previous published data using plant functional group cover, besides the study of fertilization effects where biomass was used as weight. Community N and its response to increased temperature was derived from an experiment using open-top-chambers generating an increase in summer air temperatures of 1.5 °C chambers [2] and an altitudinal gradient representing a 3 °C increase in summer air temperatures [3]. Data from the effects of herbivory was compiled from an experiment where fences excluded grazing by voles or both voles and reindeer [2]. In the fertilization experiment, reindeer feces were added at dosages corresponding to about double and four times natural abundance[4].

NDVI was derived from the same experiments and an additional experiment including warming experiments (open top chambers) from two elevations[5]. Here, previously unpublished NDVI from these studies are found in Table S3. Note that differences in NDVI between control sites, located at an altitude of 500 and 900 m.a.s.l. were used to reflect difference in greeness between these two altitudes and thus, this measure includes long-term effects.

The community-N ($N_{com.}$) was calculated as:

$$\boldsymbol{N_{com} = P_{gram} \times N_{gram} + P_{forb} \times N_{forb} + P_{everg} \times N_{everg} + P_{decid} \times N_{decid}}$$

where *P* is the relative coverage (%) for plant functional groups in subscript (gram = graminoids; forb = Forbs; everg = evergreen shrubs; and decid = deciduous shrubs) and N is the measured N content (%) of the same functional groups (in subscript).

Supplementary Table 4. **Summary of the applied statistical models.** Vegetation type (V), earthworm treatment (E), labeled litter (L) and their interactions (×) are shown along with F-values for the statistical models (GLM and LME). Statistical significance is indicated with * (P<0.05), ** (P<0.01) *** (P<0.001). Dash (-) denotes not applicable statistical test

Tested variable	E	V	L	E×V	E×L	V×L	E×V×L
%N *Festuca ovina*	$\mathbf{43.3_{1,6}}^{***}$	$0.6_{1,6}$	$0.2_{1,24}$	$\mathbf{5.6_{1,24}}^{*}$	$0.1_{1,24}$	$0.5_{1,24}$	$1.5_{1,24}$
δ15N *Festuca ovina*	$\mathbf{20.9_{1,6}}^{**}$	$2.7_{1,24}$	$\mathbf{57.1_{1,24}}^{***}$	$1.0_{1,24}$	$\mathbf{4.7_{1,36}}^{*}$	$0.2_{1,24}$	$0.2_{1,24}$
%N *Polygonum viviparum*	$5.6_{1,6}$	-	$0.0_{1,14}$	-	$2.7_{1,14}$	-	-
δ15N *Polygonum viviparum*	$0.1_{1,6}$	-	$3.4_{1,14}$	-	$0.9_{1,14}$	-	-
%N *Saussurea alpina*	$0.2_{1,6}$	-	$0.0_{1,14}$	-	$0.4_{1,14}$	-	-
δ15N *Saussurea alpina*	$\mathbf{15.1_{1,6}}^{**}$	-	$1.5_{1,14}$	-	$\mathbf{4.7_{1,14*}}$	-	-
%N *Vaccinium myrtillus*	$5.6_{1,6}$	-	$0.0_{1,14}$	-	$2.7_{1,14}$	-	-
δ15N *Vaccinium myrtillus*	$0.1_{1,6}$	-	$3.4_{1,14}$	-	$0.9_{1,14}$	-	-
%N *Vaccinium vitis-idaea*	$\mathbf{13.7_{1,6}}^{**}$	-	$\mathbf{5.6_{1,14}}^{*}$	-	$1.3_{1,14}$	-	-
δ15N *Vaccinium vitis-idaea*	$2.0_{1,6}$	-	$1.6_{1,14}$	-	$\mathbf{0.9_{1,14}}^{*}$	-	-
Soil moisture (gravimetric)	$1.0_{1,6}$	$\mathbf{9.7_{1,38}}^{**}$		$1.6_{1,38}$			
Soil moisture (volumetric)	$0.1_{1,6}$	$\mathbf{16.4_{1,9}}^{**}$		$1.7_{1,9}$			
Surface Litter	$4.7_{1,6}$	$\mathbf{79.7_{1,38}}^{***}$	-	$\mathbf{7.9_{1,38}}^{**}$	-	-	-
PLFA Actinomycetes	$1.1_{1,6}$	$\mathbf{38.7_{1,38}}^{***}$	-	$\mathbf{4.6_{1,38}}^{*}$	-	-	-
PLFA Other bacteria	$0.8_{1,6}$	$\mathbf{26.6_{1,38}}^{***}$	-	$\mathbf{4.8_{1,38}}^{*}$	-	-	-
PLFA Fungi	$0.0_{1,6}$	$\mathbf{10.4_{1,38}}^{***}$	-	$0.8_{1,38}$	-	-	-
NDVI	$\mathbf{6.4_{1,6}}^{*}$	$2.7_{1,38}$	-	$0.5_{1,38}$	-	-	-
Height Deschampsia	$4.9_{1,6}$	$\mathbf{13.1_{1,38}}^{***}$	-	$\mathbf{9.3_{1,38}}^{**}$	-	-	-
Floral shoots Deschampsia	$2.9_{1,6}$	$1.6_{1,38}$	-	$2.4_{1,38}$	-	-	-
Height Festuca	$0.1_{1,6}$	$0.5_{1,38}$	-	$0.1_{1,38}$	-	-	-
Floral shoots Festuca	$\mathbf{4.7_{1,6}}^{*}$	$\mathbf{5.6_{1,38}*}$	-	$1.5_{1,38}$	-	-	-
Total root length (start)	$0.2_{1,6}$	$0.3_{1,38}$	-	$0.1_{1,38}$	-	-	-
Root growth (after 55 days)	$1.9_{1,6}$	$2.3_{1,38}$		$\mathbf{4.5_{1,38}}^{*}$			
Root growth (after 103 days)	$\mathbf{24.2_{1,6}}^{**}$	$2.5_{1,38}$		$0.0_{1,38}$			

Supplementary Table 5. **Previously published data on the normalized difference vegetation index in response to environmental drivers.** We retrieved data from previously published studies that manipulated environmental drivers in our study area, sub-arctic northern Sweden. The altitude experiment, grazing and fertilization experiments are previously described in detail in published publications[2,3], whereas the normalized difference vegetation index (NDVI) data from the open top chambers (OTC) are in review[5]. Experimental design and vegetation type are shown along number of replicates (N), mean values (NDVI) and their standard deviation (stdev).

Experiment type	Vegetation type	Treatment	N	NDVI (mean)	stdev
**Altitude gradient**	Heath	500 m.a.s.l	5	0.81	0.03
**Altitude gradient**	Heath	900 m.a.s.l	5	0.77	0.02
**OTC**	Heath	500 m.a.s.l +1 °C	5	0.81	0.03
**OTC**	Heath	900 m.a.s.l +1 °C	5	0.76	0.02
**Grazed. control**	Heath	Vole, reindeer grazing	72	0.81	0.06
**Grazer exclusion**	Heath	No voles, no reindeer	56	0.81	0.05
**Grazer exclusion**	Heath	Vole grazing, no reindeer	70	0.80	0.06
**Fertilization (reindeer feces)**	Heath	Control (natural abundance)	10	0.78	0.01
**Fertilization (reindeer feces)**	Heath	Double natural abundance	10	0.79	0.01
**Fertilization (reindeer feces)**	Heath	High (c.4 times natural abundance)	10	0.80	0.01
**Fertilization (reindeer feces)**	Heath	Complete removal	10	0.78	0.01
**Fertilization (reindeer feces)**	Meadow	Control (natural abundance)	9	0.78	0.01
**Fertilization (reindeer feces)**	Meadow	Double	9	0.79	0.00
**Fertilization (reindeer feces)**	Meadow	High	9	0.80	0.01
**Fertilization (reindeer feces)**	Meadow	Complete removal	9	0.78	0.01

Supplementary References

1 Wackett, A. A., Yoo, K., Olofsson, J. & Klaminder, J. Human-mediated introduction of geoengineering earthworms in the Fennoscandian arctic. *Biol. Invasions* **20**, 1377-1386, doi:10.1007/s10530-017-1642-7 (2018).

2 Kaarlejarvi, E. *et al.* Effects of Warming on Shrub Abundance and Chemistry Drive Ecosystem-Level Changes in a Forest-Tundra Ecotone. *Ecosystems* **15**, 1219-1233, doi:10.1007/s10021-012-9580-9 (2012).

3 Sundqvist, M. K., Giesler, R. & Wardle, D. A. Within- and Across-Species Responses of Plant Traits and Litter Decomposition to Elevation across Contrasting Vegetation Types in Subarctic Tundra. *PLoS One* **6**, 12, doi:10.1371/journal.pone.0027056 (2011).

4 Barthelemy, H., Stark, S. & Olofsson, J. Strong Responses of Subarctic Plant Communities to Long-Term Reindeer Feces Manipulation. *Ecosystems* **18**, 740-751, doi:10.1007/s10021-015-9856-y (2015).

5 Sundqvist, M. K. *et al.* Responses of tundra plant community carbon to experimental warming, dominant species removal and elevation *Submitted manuscript* (2020).
